# Patterns of Information-Seeking for Cancer on the Internet: An Analysis of Real World Data

**DOI:** 10.1371/journal.pone.0045921

**Published:** 2012-09-21

**Authors:** Yishai Ofran, Ora Paltiel, Dan Pelleg, Jacob M. Rowe, Elad Yom-Tov

**Affiliations:** 1 Department of Hematology and Bone Marrow Transplantation, Rambam Medical Center, Haifa, Israel; 2 Bruce Rappaport Faculty of Medicine, Technion, Haifa, Israel; 3 Department of Hematology and School of Public Health, Hadassah University Hospital, Jerusalem, Israel; 4 Yahoo Research, Haifa, Israel; 5 Shaare Zedek Medical Center, Jerusalem, Israel; 6 Yahoo Research, New York, New York, United States of America; Umeå University, Sweden

## Abstract

Although traditionally the primary information sources for cancer patients have been the treating medical team, patients and their relatives increasingly turn to the Internet, though this source may be misleading and confusing. We assess Internet searching patterns to understand the information needs of cancer patients and their acquaintances, as well as to discern their underlying psychological states. We screened 232,681 anonymous users who initiated cancer-specific queries on the Yahoo Web search engine over three months, and selected for study users with high levels of interest in this topic. Searches were partitioned by expected survival for the disease being searched. We compared the search patterns of anonymous users and their contacts. Users seeking information on aggressive malignancies exhibited shorter search periods, focusing on disease- and treatment-related information. Users seeking knowledge regarding more indolent tumors searched for longer periods, alternated between different subjects, and demonstrated a high interest in topics such as support groups. Acquaintances searched for longer periods than the proband user when seeking information on aggressive (compared to indolent) cancers. Information needs can be modeled as transitioning between five discrete states, each with a unique signature representing the type of information of interest to the user. Thus, early phases of information-seeking for cancer follow a specific dynamic pattern. Areas of interest are disease dependent and vary between probands and their contacts. These patterns can be used by physicians and medical Web site authors to tailor information to the needs of patients and family members.

## Introduction

Thirty five years ago, Dr Franz Ingelfinger, a gastroenterologist and former editor of the *New England Journal of Medicine*, was diagnosed with esophageal adenocarcinoma. In a lecture delivered following his diagnosis [1], Dr. Ingelfinger described the distress caused by the flood of information aimed at him and his family. He praised one of his friends for advising him to, “forget the information you got from many quarters and look for a person who would simply tell you what to do”. “You need a doctor”, he said [1]. Back in the late seventies, doctors were the main (and for many patients the only) source of information. Today, patients need not be editors of a medical journal in order to face torrents of information gushing from the Internet. This barrage of “facts” challenges doctors' role and authority. In the case of cancer diagnosis, most patients search the Internet for cancer information, usually prior to their initial meeting with an oncology specialist [2]. Their information needs appear to differ by cancer type [3]. Internet based information was reported by patients to be a trigger for their decisions about therapy, clinical trials and even choosing their doctors. However, studies have shown that patients tend not to share informal information with their doctors [4]–[6]. Hence, a good understanding of how patients manage information acquisition through the Internet is essential for effective patient-doctor communication that will be relevant to patients' needs.

Investigating the way patients search for information on the Web is difficult. Most searches are performed in privacy away from medical facilities or health professionals. Therefore, two main ways can be used to study it: First, through retrospective questionnaires or via content generated while searching for this information on the web. Self-reported questionnaires or interviews [7], [8] have some major drawbacks. First, there is an inherent selection bias regarding patients who consent to participate in the survey, with a tendency to include people with specific sociodemographic characteristics such as higher income and education levels [9]. Second, even a low non-response rate could bias study conclusions [10]. Third, surveys usually focus on patients, though in many cases information gathering through the Internet is conducted by patient's family members [11], [12]. Last, information gathered during the first few days of illness is considered fundamental for a patient's perception of the disease [13]–[15]. However, it is very difficult to conduct surveys covering this short and stressful period [16], and retrospective self-reports have been shown to be inaccurate [17].

Search engines and other Web sites record data produced through purposeful activity (e.g., writing blogs or sending queries) and indirect activity (e.g., browsing the web). Known as User-Generated Content (UGC), these have been used to monitor the spread of influenza [18], investigate the usefulness of lithium carbonate in amyotrophic lateral sclerosis [19], and monitor seasonal and geographical changes in depression incidence [20] among other epidemiological questions [21]–[23]. In our work, we examined anonymous large scale UGC, provided by the Yahoo search engine to examine cancer-related information-seeking patterns.

## Methods

### Data collection and categorization

All queries posted by users of the Yahoo Web search engine in the USA between May and July 2010 were analyzed. Data used for conducting this study is privileged information collected by Yahoo Inc., and collated by DP and EYT, who are employees of Yahoo Inc. The information on each query included the query text, its time and date, a list of pages visited by the user as a result of the query, and an anonymized user identifier.

The queries were filtered to include only cancer-specific queries, defined as those that included the name of at least one of the 35 most common specific cancers listed by SEER [24], and divided by their 5-year survival rates into two different groups: aggressive (5-year survival below the general median for cancer patients) and indolent (5-year survival above the general median for cancer patients).

### Ethics statement

All of the research described herein was carried out according to the Yahoo guidelines on human subject research, following prior approval by Yahoo's internal Human Subject Research advisory committee. Specifically for this task, data were first anonymized and aggregated prior to analysis, and no individual-level user datum was examined. This data may be available to researchers following a signing of the necessary legal agreements to maintain user privacy.

### Identifying users with high interest in specific cancers

Among the 232,681 screened users who searched for cancer related information, most searchers viewed only scant pages. These users were more likely using Yahoo search engine as a portal to get to a specific Web-page and were excluded from our study. The distribution of the number of pages viewed by each user is highly skewed. Indeed, it is closely approximated by a power-law distribution, with slopes of −2.19 (R^2^ = 0.97), which describes a distribution pattern whereby most users view only a few pages and a minority view many pages. Therefore, an arbitrary threshold of five pages visited while posting cancer-related queries during the study period was chosen, to identify users with the strongest interest in specific cancers. In the event that users searched for more than one cancer type, the dominant disease per searcher was defined as the specific cancer type that they searched for with the highest frequency. Using the above-mentioned criteria, 182,564 users were excluded and a total of 50,117 users who posted 225,675 queries were included in the study.

### Identifying and categorization of popular web-pages

The number of users who viewed each page has a similar power-law distribution, with slopes of −2.25 (R^2^ = 0.98). To explore the content of the most popular pages, we focused only on pages visited by at least five users and manually inspected a random sample of 500 pages. Ten categories were identified, allowing classification of pages according to their themes. To label all pages visited by all users, crowd sourcing was used, specifically the Amazon Mechanical Turk service (https://www.mturk.com/), which allows each page to be shown to evaluators who are paid to categorize the Web pages [25]. Previous work has shown that this categorization is performed with accuracy comparable to that of experts [26]. Each page was sent to 5 independent people for labeling into one of the 10 categories.

All 5987 frequently visited pages were introduced to 776 Mechanical Turk labelers, each of whom labeled, on average, 34 pages (±181 pages). Of these, 3602 pages had a distinct label agreed on by at least 3 labelers. The number of pages in each category is shown in Table 1. The remaining 2385 pages did not have well-defined labels, and were divided as follows: In 46% of the pages, two categories received two votes each, and in 48% one category received two votes and three received one vote each. To explore potential reasons for disagreement between labelers, pages were in-silico quantified by analyzing the words they contained. We used a vector space model [27] to represent the words on each page, and measured the distance to the centroid (i.e., the average vector model) of each category. The distance of all non-classified pages to the categories indicated by the labelers was approximately the same (on average within 5%). However, for pages where there was a majority agreement between labelers, this difference was, on average, 20% (rank sum test, p<10^−10^). Our conclusion is that pages with non-discrete labeling are genuinely ambiguous in terms of labeling, and are thus excluded from our analysis.

**Table 1:** Categories of Web pages and the number of pages labeled within the category by Mechanical Turk.

Category	Number of pages
Causes of cancer, including environmental effects, hereditary effects, etc.	36
Preventive medicine: Self exams, vaccines against cancer	26
Symptoms of cancer	306
Information about cancer: What is cancer, new science, prognosis for various cancers and stages (hereafter denoted by “Information”)	1723
Treatment of cancer, including alternative treatment, new treatments, and drugs	305
Social media: Internet question and answer groups	133
Support organizations, awareness, and novelty items	305
News about celebrities suffering from cancer	14
Cancer of cats, dogs, and other pets	72
All other topics not covered by the categories above	435

### Recognizing search patterns

Users' search patterns were defined as the order in which they browse pages from different categories, as well as the time taken to do this. The need for information is assumed to be dynamic, changing as a function of physical and mental changes [28]. Changing patterns of the type of information requested may reflect transition between mental states common to individuals who share clinical and psychological states. These unobservable states are hidden, but may be identified from the search patterns using Hidden Markov Models (HMMs) [29], which estimate the hidden states of a system, the probability of transition between them, and the likelihood of each observable signal given the hidden state. To find the most likely number of hidden states users went through during the time we observed them, we first trained HMMs of a varying number of hidden states, and 10 visible pages categories. The best number of hidden states was found using holdout, as follows: we trained the HMM using browsing data from 75% of the users, and tested the model accuracy by comparing the predicted sequences of searches for the remaining 25% of the users with their actual behavior. This procedure was repeated 5 times with a random selection of the initial model parameter, to reduce the chance of convergence to a local minimum.

### Proband's acquaintances

Finally, in order to study the relationship between searches of social acquaintances, we used the list of contacts in the Yahoo Instant Messenger (YIM) application as a proxy for users' social network. The number of contacts per person is power-law distributed (α = −0.99, R^2^ = 0.93), with a median of 6.

### Statistical analysis

We compared the patterns of search using non-parametric tests, owing to the non-Gaussian distributions of these patterns. P values less than 0.05 were considered statistically significant.

## Results

### How likely are our seekers to be real cancer patients?

**Figure 1:**
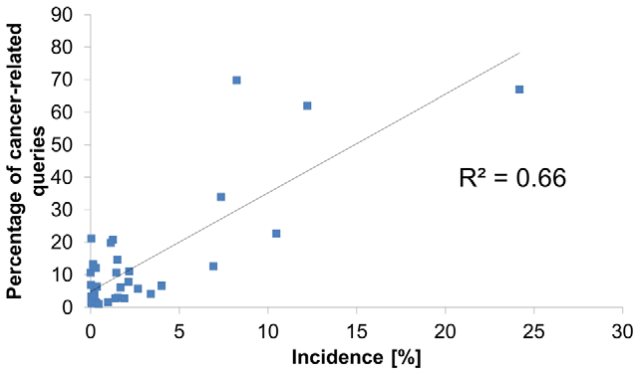
Percentage of queries for each cancer as a function of age-adjusted relative incidence. The incidence of cancers is strongly correlated with the frequency at which queries related to them are posted on the Internet.

We used a linear regression model to compare the frequency of each specific cancer type in the query log with its known age-adjusted incidence, age-adjusted 5-year relative survival, and the median age of diagnosis. Age-adjusted incidence was highly correlated with the query log prevalence, explaining over 65% of the variance (p<10^−5^), and was the only statistically significant correlate of query log prevalence (Figure 1).

This work aimed to characterize the needs and search patterns of patients and their families, friends, and other close acquaintances who use the Web as a major source of information. Users who selected pages of only one category are likely either not patients and their primary caregivers (e.g. students) or else users who prefer other sources of information than the Web and use the Internet occasionally or only when looking for specific information. In our cohort of users we identified two groups of users: one, comprising of 20,808 (41.5%) individuals, who visited pages from two or more categories (for categories definitions see below) exhibiting interest in multiple aspects of a cancer illness. The remaining 29,369 (58.5%) users visited only pages from a single category. Users who exhibit a narrow range of interests in Web-derived cancer information are not within the focus of this work, and were thus excluded from our analysis.

Users of the first group searched for information for an average period of 10.0 days (±14.5 days) during the data period. Therefore it is apparent that a specific event may have triggered their search. We speculate that these users are either newly-diagnosed cancer patients or those very close them, who serve as their “Internet agents”. We provide additional support for this below.

### Information-seeking patterns may represent underlying psychological states and are disease-related

**Figure 2:**
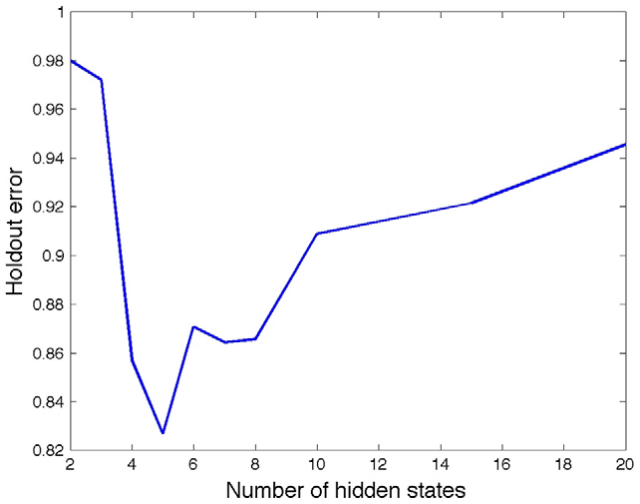
Prediction error as a function of the number of hidden states. This graph shows the average error in predicting the page category that a 25% subset of the population of users will browse, given their past browsing behavior and the model of user searches constructed using a different subset (the remaining 75%) of the population. The lowest error is reached for 5 hidden states, suggesting that users pass through five phases of search during their search process.

**Figure 3:**
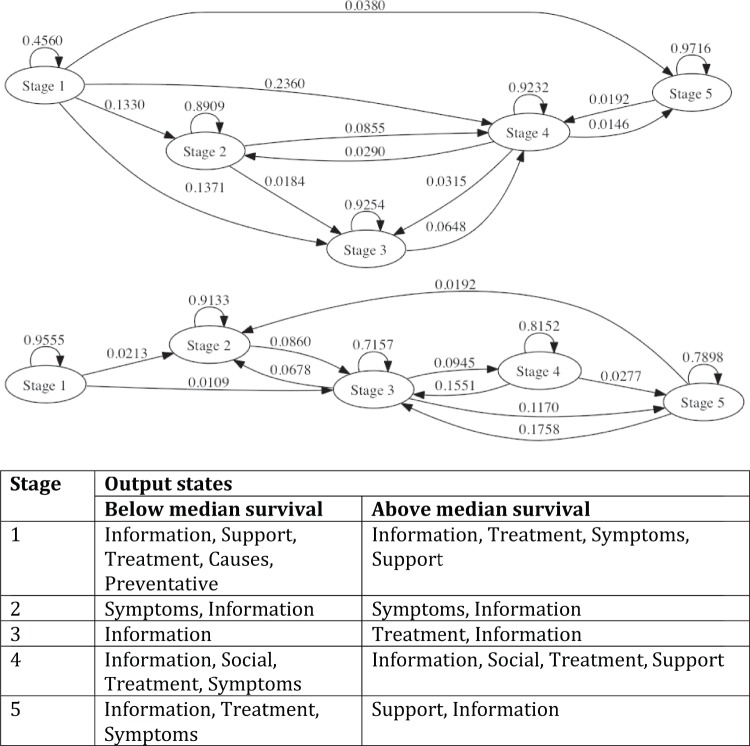
State transitions in searches for diseases with above-median (top) and below-median (middle) 5-year survival, together with the most likely output states of the HMMs (bottom). The state transitions for cancers with below-median survival are more string-like, with the first two states being the most stable states. Conversely, the state diagrams for the more indolent diseases are highly interconnected, transitions between states are more symmetric, and the most stable states are the last three states. The table shows all page categories (observable states) with a probability greater than 0.05 for each of the hidden HMM states, ordered by decreasing likelihood. While output states are similar for both groups of diseases, they are ranked differently. Furthermore, searchers for cancers with below-median survival look for treatment information later in the search process. Interestingly, support only appears as a significant output state for indolent cancers.

The most likely number of hidden states is shown in Figure 2. The best prediction of search patterns is reached with five hidden states, which we speculate are a product of transitions between five mental states during the study period. The pattern of searches was related to the aggressiveness of cancer. Figure 3 shows that the state diagram for aggressive diseases is slightly more string-like, with the first two states being the most stable, i.e., users are most likely to remain in these states. Conversely, the state diagram for the more indolent diseases are highly interconnected, transitions between states are more symmetric, and the most stable states are the last three. Comparing the HMMs learned for indolent versus aggressive diseases, we find that output states are ranked differently, and that for the latter, treatment information is more important later in the search process. Interestingly, support only appears as a significant output state for indolent cancers.

Users searching for aggressive cancers have an organized search pattern with high interest in disease information and treatment at the beginning and a low interest in supportive and social organizations. Users interested in more indolent cancers follow a less direct trajectory, moving back and forth between different categories and expressing high interest in support groups.

### Searches by acquaintances

The list of contacts of each user from Yahoo Instant Messenger (YIM) was available to identify each user's list of contacts and to analyze their relationship between cancer-related searches. During the study period, 279 users searched for cancer information in at least two categories while at least one of their contacts in YIM did so as well. We did not find a sufficiently high level of cancer-related search activity for the acquaintances of the remaining users, and thus focus on the search behavior of this relatively small group of users. We note that the number of observed pairs of simultaneously searching users is approximately 6 times greater than that expected by chance. Thus, these co-occurring searches in the network are highly significant. The type of cancer queried in at least one of the searches overlapped between proband users and acquaintances in 56% of cases. This is compared to 25% if users are randomly matched.

**Figure 4:**
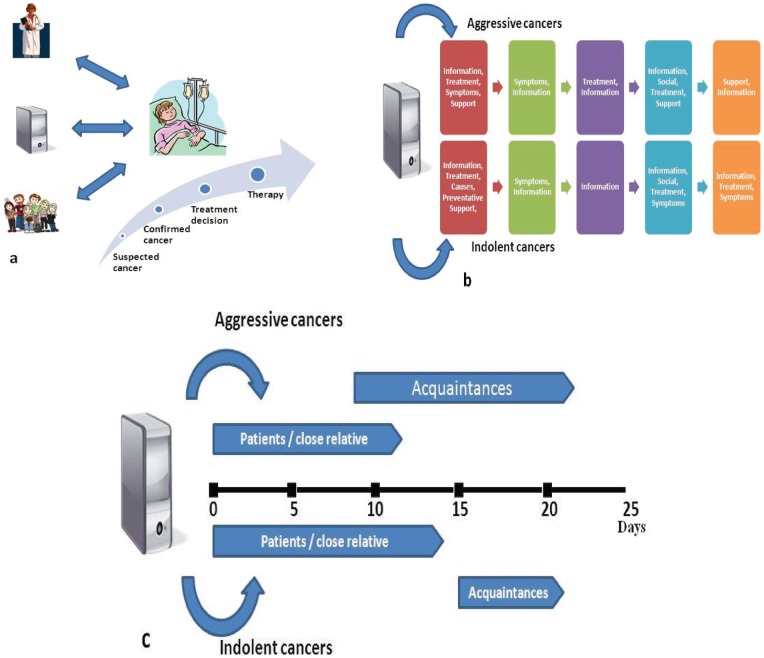
Overview of model of search pattern. a. When patients are diagnosed with cancer, they obtain information from physicians, the Internet and their friends and family. By characterizing patients information-seeking pattern we can better understand patients' needs. B. Patterns of Internet search reflect underlying psychological states. Most likely output states identified by HMMs are similar for both indolent and aggressive diseases and are represented by the colored squares. However, searchers for information on aggressive cancers look for treatment information later in the search process, while support only appears as a significant output state for indolent cancers. C. Time-line of cancer-related search by the proband seeker (most likely the patient or his primary caregiver) and other acquaintances. Acquaintances are very active searchers for aggressive diseases but much less so for indolent diseases, with a shorter search period which does not overlap with that of the initial searchers.

Acquaintances of users seeking information on more indolent diseases started searching, on average, 15 days after the first searcher. For aggressive diseases, this period was only 9 days (p = 0.06, rank sum) (Figure 4). Acquaintances searched for a much shorter period than the first user in the case of indolent disease (5.1±8.1 days versus 13.9±18.9 days, p = 0.01, sign test). In contrast, in cases of aggressive disease acquaintances searched for slightly longer than the first user (12.5±17.3 versus 11.0±14.7 days, not statistically significant). The first searcher in a pair of acquaintances looking for information on cancer searched, on average, for 28% more diseases (sign rank, p = 0.03). We hypothesize this may represent non-specific searches prior to final diagnosis.

Finally, first searchers in each pair of searchers are more likely to examine pages related to cancer treatments (25% more, p = 0.01, rank sum test) compared to second searchers) and information about cancer (14% more, not statistically significant). The later-searching acquaintances were more likely to browse pages with information on the causes of cancer (116% more (not statistically significant), compared to proband searchers) and social media related to cancer (75% more (not statistically significant)).

## Discussion

The 100 day period following cancer diagnosis is recognized as the “the existential plight in cancer” [30]. In their paper, Weisman and Worden [30] described how fundamental these first days are for coping with cancer related distress in future. Information acquired during this period shapes and defines patients' perspective about their condition.

The Internet revolutionized the way patients and their acquaintances collect information. Cancer experts often encounter patients who were drawn by the internet to erroneous self-diagnosis, unsubstantiated treatments [31], [32], or who are distressed by exposure to extreme outcome possibilities following Internet searches. In this study we focused on the information needs of presumed cancer patients and their acquaintances.

Our study shows that the information needs of intensive searchers are distinct, dependent on the severity of the cancer, and change rapidly over a period of days (Figure 4). We presume that the initial intensive searcher is the patient or close caregiver (e.g. parent, spouse). Only later other acquaintances such as friends or other family members join the search. In aggressive cancers, search time by the patient is shorter, either because of the need for urgent treatment or because patients prefer to repress numbers, statistics and facts of these diseases. Oncologists who provide reliable information, spare patients ineffective and sometime misleading Web browsing [32]. The surprisingly low proportion of Internet usage reported in parents of children with cancer and among patients with advanced stage cancers [33]–[35] may reflect a similar phenomenon. Our data may therefore reflect earlier referral to cancer experts for patients with aggressive diseases.

Patients with aggressive cancers spent a short time searching for information by themselves, however their acquaintances were involved early and searched for relatively longer periods. Conversely, for indolent cancers, acquaintances were involved late in the search process and spent only a short period searching the Internet. The findings suggest that patients and primary care-givers of patients with less aggressive tumors have more time to spend on Internet searching than those with aggressive tumors. The delay in acquaintances' searches may be due to patients' withholding information prior to the final diagnosis. A shorter delay may represent a shorter gap in aggressive cancers between early suspicion and final diagnosis or treatment decisions.

Back in 1976 independently gathering information on ones' disease was laborious and was thus very much dependent on information provided by doctors. Today, by using the internet, significant amounts of information can be gathered in very short time. Indeed our finding demonstrated that the type and intensity of searching varied within days, suggesting that in the internet era “the existential plight in cancer” lasts much less than 100 days and may vary among patients with different cancers.

Doctors see patients during a very emotionally dynamic and critical period. Patients' mental state affects the information seeking process, and the acquired information may, in turn, change the patients' mental state. Most people begin internet search prior to the meeting with the oncologist [2]. Therefore, the length of the lag between diagnosis and referral to specialists, as well as the intensity of the disease and the urgency of treatment are all likely to affect the information collecting pattern.

As we demonstrated in our results, search patterns are dependent on the severity of the disease, as is the length of the entire search process and the transition between HMM states. Strikingly, in a model of the searching patterns the number of hidden states matches the number of mental states predicted by the Kübler-Ross model [36]. However, additional research is required to demonstrate if and how the hidden HMM states match those predicted by the Kübler-Ross model (i.e., denial, anger, bargaining, depression, and acceptance), and the interplay between those internal mental states and external events.

Studies which are based on UGC, especially those in the health domain, are often the only unbiased source of information on how users seek information. As we highlighted in this study, these are essential for medical doctors to better understand their patients, and, in turn, for better information exchange between patients and caregivers. However, the need for these studies should not compromise the privacy of users. In this study we have taken specific steps to minimize this risk by anonymizing user identity and by analyzing data after aggregating it from multiple users. Thus, we believe we have gained many of the benefits of studying UGC, without compromising user privacy.

The relation between anonymous seekers and *real* cancer patients is a potential pitfall in this work. To focus on users most likely to have a close relation with a cancer patient, internet users who didn't express a wide spectrum of interest in a specific type of cancer were excluded. It is reasonable to assume that the number of such users who expressed a broad interest in a single disease for a reason other than a concrete patient is low. It should be noted that patients who didn't seek information on the web pointed to a close relative as one who surfed the Web for them [32]. Therefore, exploring the habitual searching pattern of either patients or their close relatives is important. Our study may have excluded some users who were in fact real patients if they exhibited interest in only one aspect of the disease, although they may have been represented by proxy seekers. We note, however, the strong correlation between disease prevalence and the frequency of search in the search engine, as further supporting evidence to users being actual cancer patients and their close acquaintances.

Our study has several limitations. Naive Internet users are prone to self-misdiagnosis, through a phenomenon called cyberchondria [31], when minor symptoms (eg, headache) are interpreted to be symptoms of serious diseases (eg, brain cancer). To minimize biases related to queries generated by people who are not cancer patients, only a selected group of users (42% 20,808/50,117, selected as detailed in the Methods section) was analyzed. The fact that search patterns differ by expected survival, increases the probability that we are likely dealing with real patients and their acquaintances. The Yahoo search engine was our only source of information, but only a small subset (estimated at 4%) [37] of the population are known to use multiple search engines in parallel. Users may also find information on the internet through means other than search engines. However, the data indicates that the majority of users choose search engines as the primary source of information for health-related information in general, and for cancer-related information in particular: It has been estimated that 66% of users begin their search for health-related information on search engines [38]. Moreover, a recent study found that 71% of patients who searched for cancer information primarily use a search engine as the tool of choice compared to only 13% who preferred going directly to specific cancer-related websites [2]. Information seeking using other media represents a source of information which is missing from our analysis. However, our focus on users which submitted a large number of cancer-related queries means that our data is derived from a population which uses search engines as a major source of information. The Mechanical Turk system may also be biased. Distribution of work to a large number of labelers, each of whom labeled on average only 34 pages, minimized potential biases. The data were collected during a limited time, and thus it is unknown if is the population included comprised an inception cohort. However, our findings on the length of typical search periods (10 days, compared to 90 days of data) suggest that the population that started to search before data collection began is relatively small. Furthermore, the HMM algorithm aligns data and assumes that the beginning of the search sequence may be missing. The effect of the non-inception cohort on our results is, thus, small. Although larger scale studies would obviously lend greater weight to the finding, this still represent the largest database of its kind, limited only by practical consideration (e.g., combining the data from multiple search providers or collecting data over a longer period of time). It is likely that results would be reproducible in other similarly large studies.

Notwithstanding these limitations, this study shows that UGC Internet data can be used to investigate medical questions on a large scale. Monitoring users' search patterns may help Websites to improve and tailor communication strategies to meet patients' dynamic needs, as such measures are known to improve user experience [39] ^.^ More importantly, our findings enable doctors to more fully understand the information needs of patients and highlight the importance of effective and comprehensive information transfer between doctors and their patients, from the very first day of suspicion through the process of diagnosis. By understanding the patterns of Internet use, physicians can use this tool as a powerful partner rather than a source of distress in the care of their patients. Moreover, Internet content providers need to personalize their content by taking patient search history into account when serving content, as our findings demonstrate that information need changes over time.
